# Supplementary material for: Neuronal Growth and Formation of Neuron Networks on Directional Surfaces
Source: Biomimetics (Basel). 2021 Jun 16;6(2):41. doi: 10.3390/biomimetics6020041 (PMC8293217; doi:10.3390/biomimetics6020041)
Supplement: Supplementary file 1 [file biomimetics-06-00041-s001.zip › biomimetics-1205395-si final.pdf]

Supplemental Material for

Neuronal Growth and Formation of Neuron Networks on Directional Surfaces

Ilya Yurchenko [1], Matthew Farwell[1], Donovan D. Brady[1], Cristian Staii[1,*]

1. Department of Physics and Astronomy, Tufts University, Medford, Massachusetts 02155, USA

[*] Corresponding Author: Prof. C. Staii, E-mail: Cristian.Staii@tufts.edu

Keywords: Keywords: Neuron, Axonal Growth, Neuron Networks, Neural Repair, Tissue Engineering, Stochastic Processes

To be submitted to Biomimetics

Surface preparation and cell culture

To fabricate micro-patterned substrates we start with 20mL polydimethylsiloxane (PDMS) solution (Silgard, Dow Corning) and pour it over diffraction gratings with slit separations: 1 μm - 6 μm (in increments of 1 μm) and total surface area 25 x 25 mm^2 (Scientrific Pty. and Newport Corp. Irvine, CA). The PDMS films were left to polymerize for 48 hrs at room temperature, then peeled away from the diffraction gratings and cured at 55^0 C for 3 hrs. We use AFM imaging to ensure that the pattern was successfully transferred from the diffraction grating to the PDMS surface (Figure 1). The result is a series of periodic patterns (parallel lines with crests and troughs) with constant distance d between two adjacent lines. The AFM image in Figure 1 shows that the patterns are periodic and have constant depth. The surfaces were then glued to glass slides using silicone glue and dried for 48 hours. Next, each surface was cleaned with sterile water and spin-coated with 3 mL of Poly-D-lysine (PDL) (Sigma-Aldrich, St. Louis, MO) solution of concentration 0.1 mg/mL. The spinning was performed for 10 minutes at 1000 RPM. Prior to cell culture the surfaces have been sterilized using ultraviolet light for 30 minutes.

Cortical neurons have been obtained from rat embryos (day 18 embryos obtained from Tufts Medical School). The brain tissue protocol was approved by Tufts University Institutional Animal Care Use Committee and complies with the NIH guide for the Care and Use of Laboratory Animals. The cortices have been incubated in 5 mL of trypsin at 37ºC for 20 minutes. To inhibit the trypsin we have used 10 mL of soybean trypsin inhibitor (Life Technologies). Next, the neuronal cells have been mechanically dissociated, centrifuged, and the supernatant was removed. After this step the neurons have been re-suspended in 20 mL of neurobasal medium (Life Technologies) enhanced with GlutaMAX, b27 (Life Technologies), and pen/strep. Finally, the

neurons have been re-dispersed with a pipette, counted, and plated on PDL coated glass, or PDL coated PDMS substrates, at a density of 4,000 cells/cm^2.

Fluorescence and AFM Imaging

For fluorescence imaging the cortical neurons cultured on glass or PDMS surfaces, were rinsed with phosphate buffered saline (PBS) and then incubated for 30 minutes at 37ºC with 50 nM Tubulin Tracker Green (Oregon Green 488 Taxol, bis-Acetate, Life Technologies, Grand Island, NY) in PBS. The samples were then rinsed twice with PBS and re-immersed in PBS solution for imaging. Fluorescence images were acquired using a standard Fluorescein isothiocyanate -FITC filter: excitation of 495 nm and emission 521 nm. We have previously shown that both untreated and chemically modified neurons grown in the MFP3D fluid cell remain viable over long periods of time [10, 16, 19, 20, 30-32]. Axon outgrowth was tracked using ImageJ (National Institute of Health). To obtain the angular distributions (Figure 3, Figure S2) all axons have been tracked and then partitioned into segments of 20 μm in length. We have then recorded the angle that each segment makes with the *x* axis (Figure 1(b)), and the results were plotted as angular histograms (Figures 3 and S2). All surfaces were imaged using an MFP3D Atomic Force Microscope (AFM), equipped with a BioHeater closed fluid cell, and an inverted Nikon Eclipse Ti optical microscope (Micro Video Instruments, Avon, MA). The AFM topographical images of the surfaces were obtained using the AC mode of operation, and AC 160TS cantilevers (Asylum Research, Santa Barbara, CA). Surfaces were imaged both before and after neuronal culture, and no significant change in topography was observed.

Simulations of growth cone trajectories

We perform simulations of growth cone trajectories using the stochastic Euler method with N steps [26,27]. With this method the change in position of the growth cone and the turning angle at each step are parametrized by the arclength s from the axon's initial position:

$$\Delta x(s) = \cos(\theta) \cdot \Delta s$$

$$\Delta y(s) = \sin(\theta) \cdot \Delta s$$

$$\Delta\theta(s) = -\gamma_\theta \cdot \cos(\theta) + D_\theta \cdot dW$$

where $-\gamma_\theta \cdot \cos(\theta)$ is a deterministic steering torque, and $D_\theta \cdot dW$ is an uncorrelated Wiener process representing the randomness in the axon steering (γ_θ and D_θ represent the damping and diffusion coefficients, respectively, which are defined in the main text, see Equation (1)). The angle θ is determined from the angular probability distribution (Equations (1) and (2) in the main text). The velocity distributions are obtained from the change in position of the growth cone at each step [26,27].

Additional Experimental Data

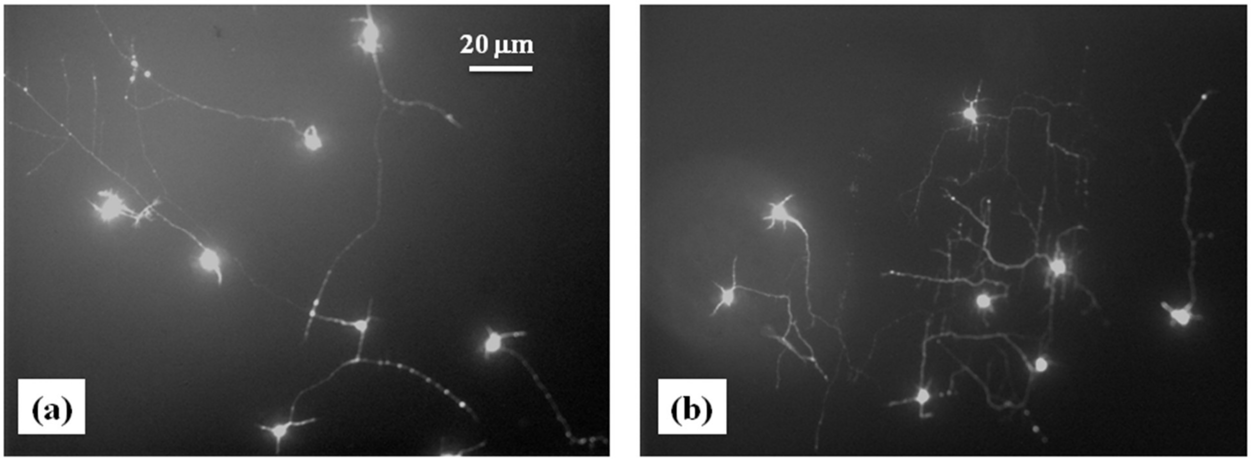

Figure S1. Fluorescence (Tubulin Tracker Green) images showing examples of axonal growth for cortical neurons treated with Y-27632, a chemical compounds that inhibits the dynamics of actin filaments. The images are captured 36 hrs after neuron plating. The scale bar shown in (a) is the same for both images. The pattern spatial period is d =3 μm in (a), and d =5 μm in (b).

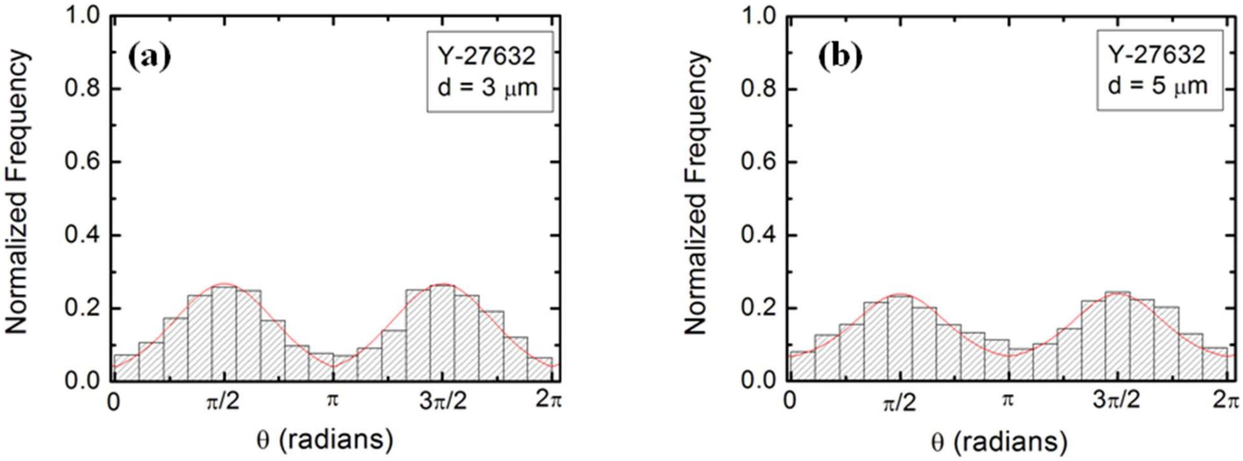

Figure S2. Examples of normalized experimental angular distributions for axonal growth measured on micropatterned PDMS surfaces for neurons treated with Y-27632. The vertical axis (labeled Normalized Frequency) represents the ratio between the number of axonal segments growing in a given direction and the total number N of axon segments. Each axonal segment is of

20 μm in length (see Section 2 on Data Analysis in the main text). All distributions show data collected at t =36 hrs after neuron plating. (a) Angular distribution obtained for N = 639 different axon segments for neurons treated with Y-27632 and cultured on surfaces with d =3 μm (corresponding to Figure S1(a)). (b) Angular distribution obtained for N = 604 different axon segments for neurons treated with Y-27632 and cultured on surfaces with on d =5 μm (corresponding to Figure S1(b)). The neurons treated with Y-27632 show a significant decrease in the degree of alignment with the surface patterns, compared to the untreated cells. The continuous red curves in each figure are the predictions of the theoretical model (see Section 4 in the main text).

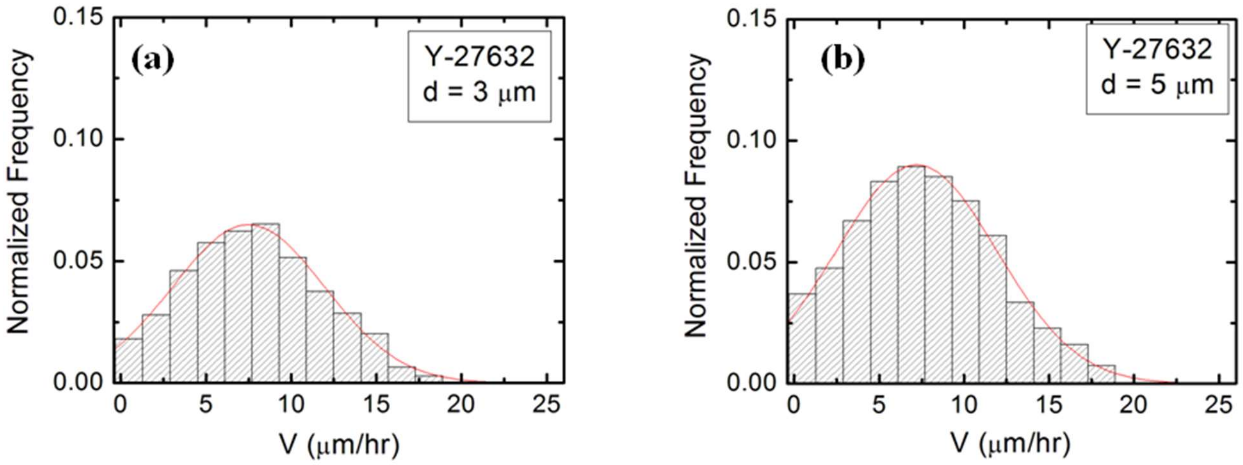

Figure S3. Examples of normalized speed distributions obtained for growth cones of cortical neurons treated with Y-27632. The growth substrates are PDL coated PDMS surfaces with periodic micropatterns with the pattern spatial period d =3 μm (a) and d =5 μm (b). The images are captured at t =36 hrs after neuron plating. (a) Speed distribution measured for N = 130 different growth cones for neurons treated with Y-27632. (b) Speed distribution measured for N = 152 different growth cones for neurons treated with Y-27632. The continuous red curves in each figure represent the predictions of the theoretical model discussed in Section 4 in the main text.

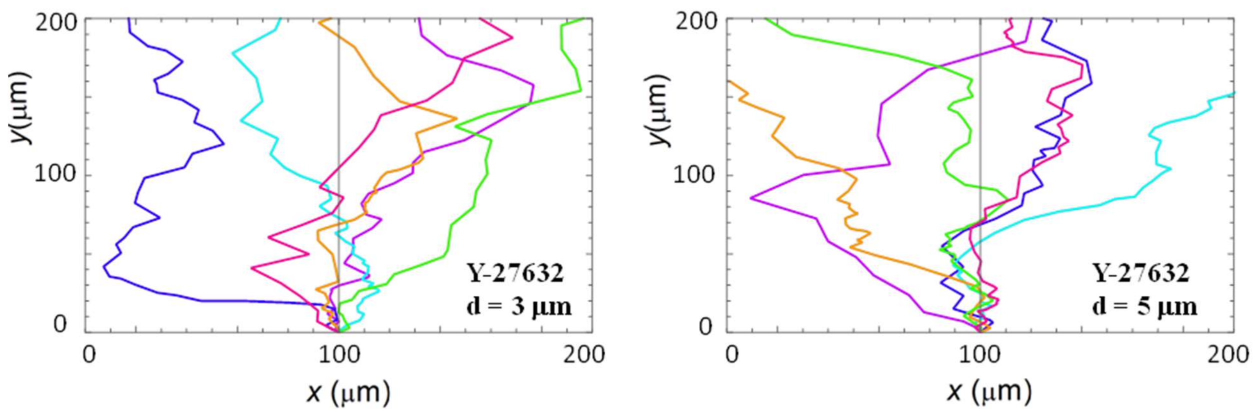

Figure S4. Examples of simulated neuronal growth for Y-27632 - treated neurons. The simulations are performed by using the values of the growth parameters obtained from the fit of the experimental data with Equations (2) and (4) (seen main text). The pattern spatial periods correspond to the data shown in Figures S1, S2 and S3: d =3 μm for (a), and d =5 μm for (b).

**Cell/Substrate**	$\gamma_\theta(\mathrm{h}^{-1})$	$D_\theta \cdot 10^{-3}(\mathrm{h}^{-1})$	$\beta(\mu\mathrm{m})^{-1}$
Untreated *d=3* μm	0.16 ± 0.02	81 ± 5	1.2 ± 0.4
Untreated/ *d=5* μm	0.18 ± 0.03	87 ± 3	1.2 ± 0.4
Taxol/ *d=3* μm	0.11 ± 0.02	53 ± 4	0.6 ± 0.3
Taxol/ *d=5* μm	0.13 ± 0.03	61 ± 5	0.6 ± 0.3
Y-27632/ *d=3* μm	0.05 ± 0.02	39 ± 2	0.4 ± 0.3
Y-27632 *d=5* μm	0.07 ± 0.02	44 ± 3	0.4 ± 0.3

Table S1. Values for the growth parameters of untreated and chemically modified neuronal cells, grown on different types of micropatterned PDMS substrates. The uncertainty for each parameter represents the uncertainties from the fit of the corresponding data points.
